# A Method for Estimating Urban Background Concentrations in Support of Hybrid Air Pollution Modeling for Environmental Health Studies

**DOI:** 10.3390/ijerph111010518

**Published:** 2014-10-15

**Authors:** Saravanan Arunachalam, Alejandro Valencia, Yasuyuki Akita, Marc L. Serre, Mohammad Omary, Valerie Garcia, Vlad Isakov

**Affiliations:** 1 Institute for the Environment, University of North Carolina at Chapel Hill, 100 Europa Drive, Suite 490, Chapel Hill, NC 27517, USA; E-Mails: valenal@email.unc.edu (A.V.); omary@email.unc.edu (M.O.); 2 Department of Environmental Sciences and Engineering, University of North Carolina at Chapel Hill, Michael Hooker Research Center, 1305 Dauer Drive, Chapel Hill, NC 27599, USA; E-Mails: akita@email.unc.edu (Y.A.); marc_serre@unc.edu (M.L.S.); 3 National Exposure Research Laboratory, U.S. Environmental Protection Agency, 109 T.W. Alexander Drive, Research Triangle Park, NC 27711 USA; E-Mails: Garcia.Val@epa.gov (V.G.); Isakov.Vlad@epa.gov (V.I.)

**Keywords:** air quality model, human exposure, background concentration, kriging, STOK, on-road emissions, traffic, NO_x_, PM_2.5_

## Abstract

Exposure studies rely on detailed characterization of air quality, either from sparsely located routine ambient monitors or from central monitoring sites that may lack spatial representativeness. Alternatively, some studies use models of various complexities to characterize local-scale air quality, but often with poor representation of background concentrations. A hybrid approach that addresses this drawback combines a regional-scale model to provide background concentrations and a local-scale model to assess impacts of local sources. However, this approach may double-count sources in the study regions. To address these limitations, we carefully define the background concentration as the concentration that would be measured if local sources were not present, and to estimate these background concentrations we developed a novel technique that combines space-time ordinary kriging (STOK) of observations with outputs from a detailed chemistry-transport model with local sources zeroed out. We applied this technique to support an exposure study in Detroit, Michigan, for several pollutants (including NO_x_ and PM_2.5_), and evaluated the estimated hybrid concentrations (calculated by combining the background estimates that addresses this issue of double counting with local-scale dispersion model estimates) using observations. Our results demonstrate the strength of this approach specifically by eliminating the problem of double-counting reported in previous hybrid modeling approaches leading to improved estimates of background concentrations, and further highlight the relative importance of NO_x_ *vs.* PM_2.5_ in their relative contributions to total concentrations. While a key limitation of this approach is the requirement for another detailed model simulation to avoid double-counting, STOK improves the overall characterization of background concentrations at very fine spatial scales.

## 1. Introduction

Through the Clean Air Act (CAA), the U.S. Environmental Protection Agency (EPA) develops air quality standards to protect the public from the health effects of criteria air pollutants (ozone, carbon monoxide, oxides of nitrogen, particulate matter, lead, and oxides of sulfur) and hazardous air pollutants (HAPs). These Congressional mandates have led to a systematic risk assessment approach that encompasses hazard identification, dose-response assessment, exposure assessment, and risk characterization [1]. As this field of risk assessment has evolved, so has the reliance on epidemiological studies for identifying hazards due to air pollutants; quantifying the relationship between dose, exposure or concentration, and the response; and determining and assessing mitigation strategies [1,2,3]. 

In the absence of personal exposure measurements, epidemiological studies have traditionally relied upon alternative indicators of exposure, such as area-wide ambient air pollution concentrations from central monitoring sites. These studies assume that concentrations at a single monitor, or average concentrations over a few monitors, are representative of the complex spatial and temporal patterns of air quality within a study area. However, there is increasing evidence that the monitoring network is not capturing the sharp gradients in exposure that can occur in areas with high concentrations (e.g., near major roadways) [4]. To reduce uncertainty that may be introduced via exposure misclassification, these epidemiological studies (especially time-series studies) require an accurate assessment of the complex temporal and spatial variations in ambient concentrations [1]. The impact of exposure misclassification on the outcome of air pollution epidemiological studies varies depending on the particular study design [5]. In general, finer spatial and temporal resolutions will decrease exposure misclassification. This is particularly relevant for those pollutants that exhibit strong gradients or are heterogeneous across space. Therefore, models representing the local- and regional-scale features of emissions can be used to better characterize exposure [6,7,8]. In addition to comparing hybrid model concentrations (with and without local emissions using the “zero-out” approach), we used a Space-Time Ordinary Kriging (STOK) model to estimate the background contribution from regional transport and photochemical transformations, and compared the resulting hybrid model concentration estimates with available ambient monitoring data.

There are several available modeling approaches capable of providing spatially and temporally resolved air pollutant concentration at a fine resolution [8,9,10,11,12,13,14]. These can be categorized into two major types of air quality models: regional photochemical grid models and local-scale dispersion models:

- Regional photochemical grid models, such as the Community Multiscale Air Quality (CMAQ) model [15], are used to simulate the transport and formation of ozone, acid rain, PM_2.5_, and other pollutants formed by chemical reactions among precursor species that are emitted from hundreds or thousands of sources. CMAQ provides volume-averaged average hourly concentration values for each grid cell in the modeling domain. Emissions are assumed to be instantaneously well-mixed. Models such as CMAQ are usually applied over a wide range of spatial scales ranging from national (thousands of kilometers) to urban (a few kilometers). However, these models can address neither the near-source gradients nor the local-scale (10–300 m) processes affecting pollutant gradients near sources such as major roadways.
- Local-scale dispersion models such as AERMOD [16] are designed to capture near-source concentration gradients (e.g., within a few kilometers from the source) and can provide detailed resolution of the spatial variations in hourly average concentrations. However, they do not take into account atmospheric chemical reactions, except for highly simplified representations such as first-order pollutant decay.

To address the limitations of these two types of models, Isakov *et al*. combined the capabilities of both into a hybrid modeling approach [8]. Concentrations from a grid-based chemistry-transport model and a local-scale dispersion model are added to provide contributions from photochemical interactions, long-range (regional) transport, and details attributable to local-scale dispersion.

A major advantage of this hybrid approach is that it integrates modeled concentrations simulated at varying spatial and temporal scales (e.g., background and near-source) to account for total emissions. By accounting for both regional and local-scale influences, the approach enables improved comparison against monitoring data at urban scales. However, a significant concern with the hybrid modeling approach has been that the same emission sources may be included in both types of models, which could result in double-counting the concentrations/impacts of these sources. In one study, Stein *et al*. qualitatively compared the hybrid approach for benzene concentrations in Houston, TX, and concluded that the emissions double-counting was about 10% and thus had no noticeable impact on the hybrid model estimates [17]. However, the magnitude of double-counting will depend on the number of sources involved as well as their distribution relative to the “regional” contribution.

This paper examines a method that both estimates the effect of double-counting on hybrid model concentrations and addresses this problem. The double-counting comes from the fact that if we are not careful, the background concentration may include local sources that are also included in the local-scale model. Local sources are usually located in urban areas of interest for epidemiologic or risk assessment studies, and therefore this issue of double-counting often arises in urban areas. To address this issue of double counting in a representative setting, we select a case study where local sources are located in an urban area of interest, and we carefully define background concentrations as the background concentration that would be measured if local sources in that urban area were zeroed out. To obtain information on urban background concentrations, we used two regional model simulations: one for the base case, in which all emission sources are included; and one in which the emissions used in the local-scale model are excluded, which we refer to as the “zero-out” approach that estimates background concentrations. The difference in concentrations between these two simulations provides a quantitative estimate of the magnitude of any emissions double-counting and its impacts on total hybrid model estimates.

In addition to comparing hybrid model concentrations with and without local emissions, we also compare the resulting hybrid model concentration estimates with available ambient monitoring data. Evaluation is critical for any air quality model applications. However, evaluating spatially and temporally resolved model concentrations in a large urban area is a challenge because observations are not usually available at these scales. In this study, we focus on Detroit, Michigan, a large urban area where we used air quality models to provide inputs for epidemiological analyses in support of the Near-Road Exposure and Effects of Urban Air Pollutants (NEXUS) study [18]. The NEXUS study was designed to examine the relationship between near-roadway exposures to several air pollutants and respiratory outcomes in a cohort of asthmatic children who live close to major roadways in Detroit. For our work, we focused on two key pollutants: NO_x_, an example of an important pollutant related to mobile sources, and PM_2.5,_ an example of a major local- and regional-scale criteria pollutant of concern. Details on the air quality modeling results that used the background concentrations from this study and extensive model evaluation using both U.S. EPA Air Quality System (AQS) routine monitoring data and data from special monitoring during the study period are described in a companion paper [19]. Additional analyses of the exposure metrics computed from these results are presented in another companion paper [20].

## 2. Estimating Background Concentrations

To estimate the background contribution from regional transport and photochemical transformations, we used a combination of a statistical model and the CMAQ photochemical grid-based model. While the overall modeling for NEXUS included the period January 2010–May 2012, in this manuscript we focus the description of our methodology and results on the year 2010 only, for the sake of illustrating the method with an annual dataset.

### 2.1. Air Quality Observations

We obtained ambient air quality monitoring concentration datasets for AQS locations in Michigan, Indiana, Ohio, and Pennsylvania from January 2009 to December 2011. Monitoring sites in multiple states beyond the study region in Detroit were included to provide a minimum sample size for the statistical approach that is discussed below. Ninety-two unique monitoring locations were identified as relevant, and we were able to use NO_x_ and PM_2.5_ data from 64 of these sites (see Table S1 in Section 1 of the Supplementary Material for a full list of the 64 sites). The AQS monitors have been classified by EPA as having one of five objectives: “*highest concentration*”, “*population exposure*”, “*source impact*”, “*general background*/*regional transport*”, and “*welfare-related*” [21]. Of these, the “general background” objective includes sites that are intended to capture two spatial scales of interest: either “*urban*” (city-wide conditions, with dimensions on the order of 4–50 km) or “*regional*” (rural area of reasonably homogeneous geography, with extents from tens to hundreds of kilometers). We leveraged this information in the AQS database to create two groups of monitors (see Table S1): one group contained all sites with an objective of “background” and the other contained the rest of the 64 sites (which we call “nonbackground” sites).

### 2.2. CMAQ Modeling

In addition to the monitoring data, we used output concentrations from two annual simulations from CMAQ v4.7.1 for the year 2005. The modeling domain covered the eastern half of the contiguous U.S. using a 12-km × 12-km horizontal grid resolution. In the baseline simulation, referred to as CMAQ_Total_, all emission sources (natural and anthropogenic) in the entire domain were modeled; this included anthropogenic emissions from all source sectors (point, area, on-road mobile, off-road mobile, dust, fire) based upon a 2006 projected year inventory from EPA’s National Emissions Inventory (NEI), and biogenic emissions from a combination of BEIS-3 and the Model of Emissions of Gases and Aerosols from Nature (MEGAN). The model configuration for this application and the evaluation of model outputs against observed datasets are described elsewhere [22]. In the second simulation, referred to as CMAQ_ZeroOut_, all local sources in the Detroit metropolitan area were zeroed-out_._ These included major and minor point sources, on-road and off-road sources, and other stationary sources that are usually treated as area sources. To perform this zero-out simulation, we first processed these local source inventories through the Sparse Matrix Operator Kernel Emissions (SMOKE) modeling system [23], performed chemical speciation and temporal and spatial allocation, and gridded to the 12-km × 12-km resolution modeling domain. We then subtracted these gridded emissions from the CMAQ_Total_ emissions files to create the model-ready CMAQ_ZeroOut_ scenario, and performed the CMAQ simulation again for the year 2005.

### 2.3. Space-Time Ordinary Kriging (STOK)

Dispersion models provide a good description of the sharp gradients in air pollution concentrations resulting from some local sources (LS) of interest. The concentration resulting from all other (nonlocal) sources is referred to as the background concentration. Because dispersion processes are in large part additive, the total concentration, Z, can be approximated as the sum of the local-source concentration, *Z_LS_*, obtained from a dispersion model, and the background concentration, *B*, obtained from a regional chemistry-transport model such as CMAQ when *local sources have been zeroed out*, *i.e.*, Z = *Z_LS_* + *B.* In this work we focus on estimating the background concentration *B*.

Because the background and total concentrations vary across space and time, let us define as *B*(***p***) and *Z*(***p***) the space/time random fields (S/TRF) representing background concentration and total concentration at a specific point ***p*** in space ***s*** and time t, ***p*** = (***s***, *t*), respectively. By convention, lower-case variables will denote realizations or deterministic values taken by their corresponding upper-case random variables. Our method relies on developing a geostatistical framework that uses concentration data collected at AQS monitoring stations to estimate background concentration at *unmonitored* locations. Because of their ability to produce not only an estimate at an unmonitored location but also the uncertainty associated with that estimate, geostatistical methods have been widely used in air quality studies. Here, we employ the method referred to as space/time ordinary kriging (STOK) with measurement error [24,25]. 

At space/time points ***p**_h_* corresponding to AQS *background* monitoring stations located away from the local sources, it is reasonable to assume that the total concentrations observed, *z*(***p**_h_*), correspond entirely to the background concentrations, *b*(***p**_h_*). Hence we treated the concentrations observed at AQS background stations located away from the local sources as *hard* data (*i.e.*, exact measurements) for the background concentration in our framework. 

In addition to the observed concentrations at monitors designated as background, we also used observed concentrations at nonbackground monitors to inform our modeling of total background concentration levels. We obtained soft data on background concentrations (space/time points ***p**_s_* corresponding to nonbackground AQS stations) by multiplying the observed total concentration, *z*(***p**_s_*), by a random variable, *R*_Background/Total_, representing the ratio of background to total concentration. This random variable is assumed to be normally distributed with a mean *μ_R_* and variance *σ*^2^*_R_* obtained from the output of the two CMAQ simulations (CMAQ_Total_ and CMAQ_ZeroOut_). The ratio CMAQ_ZeroOut_/CMAQ_Total_ is calculated for each hour of the study period, after removal of outliers lower than 1% and higher than 99%; and *μ_R_* and variance *σ*^2^*_R_* are obtained for a given nonbackground station as the mean and variance of the hourly ratios CMAQ_ZeroOut_/CMAQ_Total_ at that station. The random variable *R*_Background/Total_ therefore captures the variability and uncertainty associated with the background-to-total concentration ratio at a given nonbackground station. Since at points ***p**_s_* we have *B*(***p**_s_*) = *z*(***p**_s_*) *R*_Background/Total_, then *B*(***p**_s_*) at a given nonbackground station is given by a soft datum with mean *z*(***p**_s_*)*μ_R_* and having a measurement error with known variance *z*(***p**_s_*)^2^*σ*^2^*_R_*. Finally the hard data at location ***p**_h_* and the soft data at locations ***p**_s_* are combined to constitute the overall data *b*(***p***) available in this study.

The S/TRF *B*(***p***) describing background concentrations was defined as the sum of a homogenous/stationary S/TRF *X*(***p***) and an additive constant (or offset) *o_B_* calculated by taking the average of all the observed background concentrations. The procedure we use is as follows: We first defined the transformed data *x* using a transformation of the *b*(***p***) data written as *x*(***p***) = *b*(***p***) − *o_B_*. We then define *X*(***p***) as a homogenous/stationary S/TRF for which the transformed data *x* is a realization. The S/TRF *X*(***p***) therefore represents the variability and uncertainty associated with the transformed data *x* obtained by subtracting the additive constant *o_B_* from the data *b*(***p***). 

The STOK method was applied on the transformed data as follows: First, a three-structured space/time exponential covariance model was used to characterize the space/time autocorrelation in the transformed data. The background concentration at a given unmonitored location was then estimated by obtaining the estimate of transformed data using the STOK model and adding back the constant offset to it. We used Matlab R2010a (MathWorks Inc., Natick, MA, USA) and *BMElib2.0b* [26] for the geostatistical estimation. The covariance model, and the two components for each of NO_x_ and PM_2.5_, are provided in Table S2, Section 2 of the Supplementary Material.

## 3. Application in Detroit, Michigan

**Figure 1:**
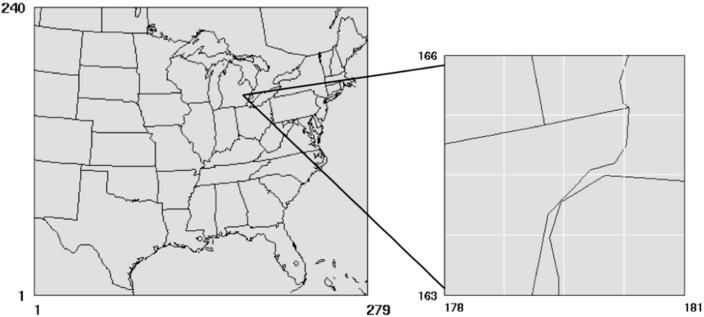
CMAQ modeling domain for the Eastern U.S. (left) and Detroit study domain (right). The Detroit grid shows the 16 12-km × 12-km grid cells where local sources were removed.

To illustrate an application of the hybrid modeling approach, we focus on a 40-km × 30-km area in Detroit, Michigan. The modeling domain includes many stationary sources, such as power plants, large ports and marine terminals, and several major roadways, such as Interstates 75, 94, and 96. In this study, these major roadways were divided into several thousand roadway links that were then modeled in the Research LINE (R-LINE) model [27] as line sources emitting at ground level. The emission factors for the mobile sources were based upon the Motor Vehicle Emissions Simulator (MOVES) model version 2010b [28]. Figure 1 shows the CMAQ regional-scale modeling domain and the zoomed-in portion for the focus area in Detroit. Our selected area encompasses the majority of the emission sources in the Detroit area. In 2010, there were four monitoring sites in the Detroit domain that provided hourly average concentrations of PM_2.5_ and one monitoring site for NO_x_.

**Figure 2:**
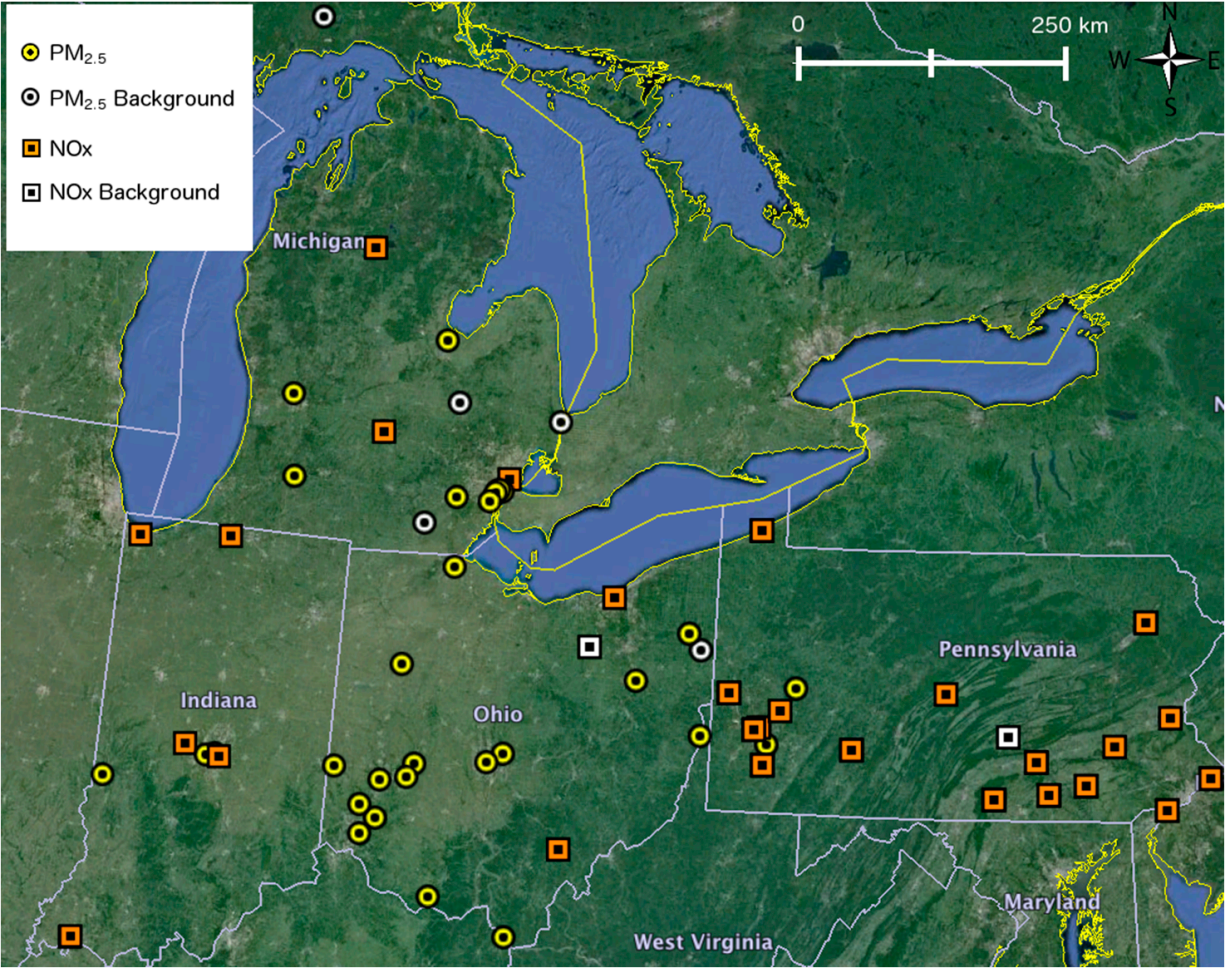
Location of AQS monitors used in the STOK algorithm.

**Figure 3:**
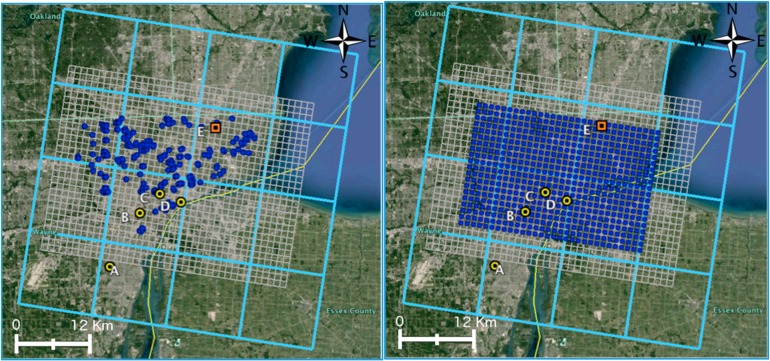
The cyan grid (CMAQ 12-km × 12-km grid) shows the 16 cells where local sources were removed. The grey grid (40 × 30 Detroit 1-km × 1-km grid) shows the location of the mobile and stationary emission sources. The blue circles indicate NEXUS (**left**) and Detroit grid (**right**) receptor locations. Yellow AQS sites labeled A through D represent PM_2.5_ sites 261630001 (Deer Park), 261630033 (Dearborn), 261630038 (Newberry), and 261630039 (Ambassador Bridge), respectively. The orange AQS site labeled E represents NO_x_ site 261630019 (E 7 Mile Road). The yellow line shows the U.S.-Canada border.

For the purpose of calculating STOK background estimates for the year 2010, we used AQS measurements from 2009, 2010, and 2011 for PM_2.5_ and NO_x_ (the STOK algorithm requires data for one year prior to and one year after the year of analysis). These sites were then classified into background sites and nonbackground sites (Figure 2). Since background concentration is defined as the concentration that would be measured if local sources in Detroit were zeroed out, we selected background sites that were at least 60 km away from Detroit so that it is reasonable to assume that the concentration observed at these stations would be the same as if local sources in Detroit were shut down. We used STOK to estimate the hourly background concentrations during 2010 for PM_2.5_ and NO_x_ at three sets of estimation locations: 30-km × 20-km Detroit grid with receptors every 1 km; residential addresses of NEXUS participants (for use in the NEXUS study, and with adequate obfuscation for privacy reasons); and AQS monitoring locations (for model evaluation). Figure 3 shows the modeling domain with source regions, AQS monitoring locations, and the two sets of receptors used in the study. 

## 4. Results and Discussion

**Figure 4:**
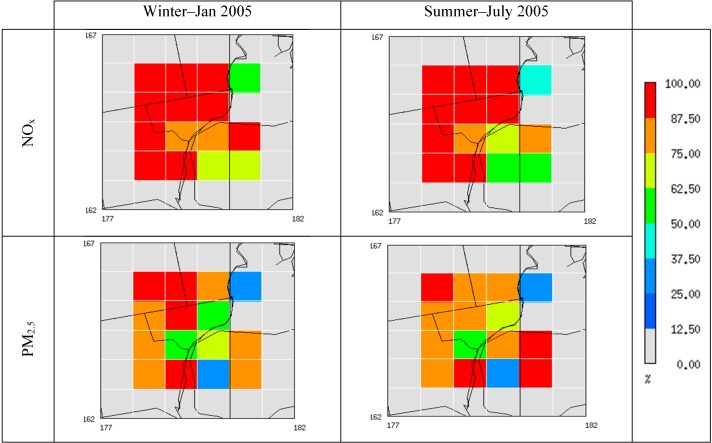
Percent reduction spatial plots ((Emission_Total_ – Emission_ZeroOut_)/Emission_Total_) for NO_x_ and PM_2.5_ for summer and winter at the 16 cells where local emissions were removed. (An extra ring of grid-cells is shown outside the 16 cells to confirm that emissions outside the Detroit study region were not modified.)

**Figure 5:**
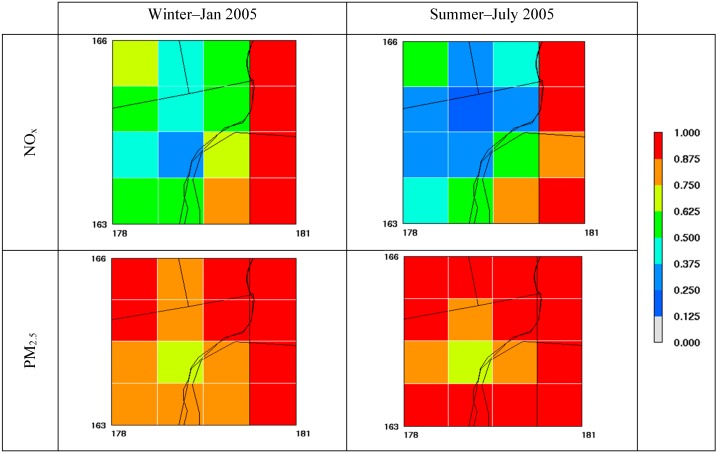
Spatial plots of CMAQ-based R_ZeroOut/Total_ for NO_x_ and PM_2.5_ for summer and winter at the 16 cells where local emissions were removed.

Before estimating background concentration using the STOK method, we evaluated the main components introduced into STOK. For example, Figure 4 shows the surface-layer emission reduction in percent due to removal of local sources in the Detroit area. We focused on our 16 cells that span across the Detroit metropolitan region, including portions of Wayne, Macomb, and Oakland Counties; it is at these locations that local sources were removed in the CMAQ_ZeroOut_ scenario. NO_x_ and PM_2.5_ were averaged for a representative summer (July) and winter (January) month in 2005 for illustrating seasonal variability in the emissions. In the Detroit counties, both NO_x_ and PM_2.5_ show reduction above 50%, whereas emission reductions in Canada (Southeast quadrant of the domain) show more variability, ranging from close to 0% to 100%. These significant emission reductions are reflected in the CMAQ simulations output shown in Figure 5. This figure shows the ratio of CMAQ_ZeroOut_ over CMAQ_Total_ (R_ZeroOut/Total_) for NO_x_ and PM_2.5_ concentrations averaged for the same representative summer and winter months. R_ZeroOut/Total_ values range from around 1 to very close to 0. R_ZeroOut/Total_ values for PM_2.5_ that are closer to 1 demonstrate that background PM_2.5_ concentrations account for the bulk of the total PM_2.5_ concentrations. NO_x_, on the other hand, shows lower R_ZeroOut/Total_ values than PM_2.5_, indicating that most of the total NO_x_ concentration is due to local anthropogenic sources. 

**Figure 6:**
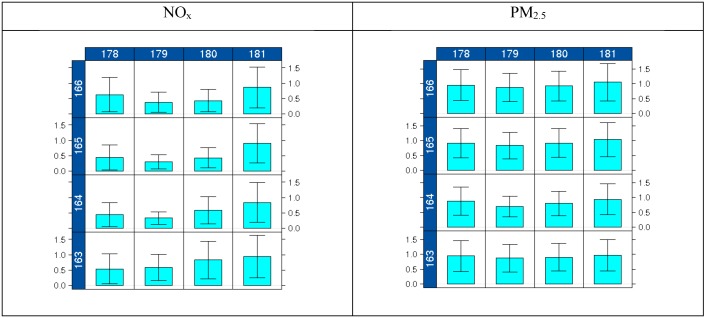
Annual mean and variance of R_ZeroOut/Total_ for NO_x_ and PM_2.5_ at the 16 cells where local emissions were removed.

Figure 6 shows the annual mean and variance in time of R_ZeroOut/Total_ for both NO_x_ and PM_2.5_; these are direct inputs to the soft data (air quality observations from nonbackground sites adjusted using R_ZeroOut/Total_) used in the STOK method. Once again, as seen in previous figures, most of the reductions are concentrated around the Detroit metro area (central cells). Reductions show means of R_ZeroOut/Total _ as low as 0.3 for NO_x_ and 0.8 for PM_2.5_. This area also depicts lower temporal variability compared to the surrounding cells, with variances of R_ZeroOut/Total_ as low as 0.03 for NO_x_ and 0.1 for PM_2.5_. 

**Figure 7:**
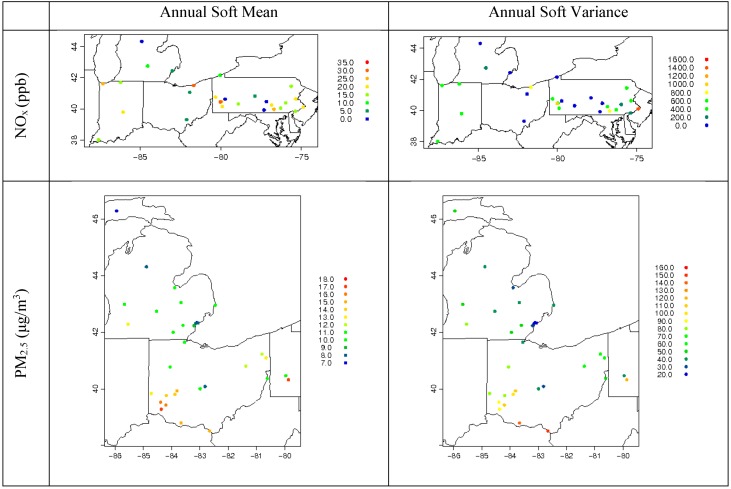
Annual soft mean and variance for NO_x_ and PM_2.5_ at all available monitors used for STOK estimation.

**Figure 8:**
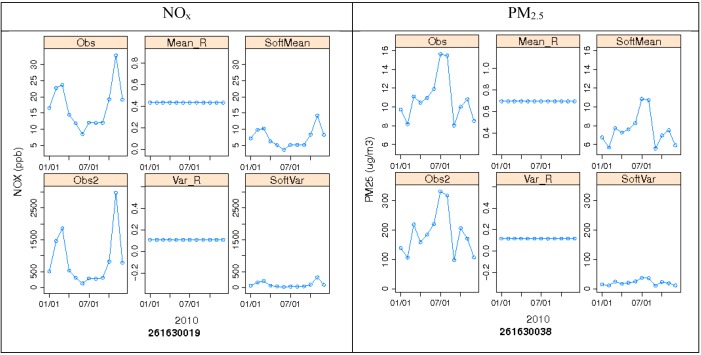
Observed concentration, R_ZeroOut/Total_ mean, R_ZeroOut/Total_ variance, soft mean, and soft variance averaged monthly at nonbackground monitors 261630019 (E 7 Mile Rd) for NO_x_ and 261630038 (Newberry) for PM_2.5_.

R_ZeroOut/Total_ means and variance in time obtained from the 2005 CMAQ simulation are then paired in space to the corresponding AQS 2010 hourly measurements to calculate soft mean *z*(***p**_s_*)*μ_R_* and soft variance *z*(***p**_s_*)^2^*σ*^2^*_R_* for every hour, using methods discussed earlier. The annual averages of the resultant soft mean and variance for NO_x_ and PM_2.5_ are shown in Figure 7. The spatial extent of the domains for NO_x_ and PM_2.5_ differ because a different set of monitors was used from the surrounding states to capture the minimum required number of monitors with valid data for use in the algorithm. Figure 7 further shows the spatial gradient of the soft data. As was expected, low soft means are depicted within the Detroit metro area. Nonetheless, the range within all monitors varies significantly, from 0 to 32 ppb for NO_x_ and from 7 to 18 μg/m^3^ for PM_2.5_. Soft variance values behave in a similar way, where lower variance is shown in the Detroit area. Figure 8 shows how observed data, CMAQ data (R_ZeroOut/Total_), and soft data mean and variance behave temporally (using monthly averages) at specific nonbackground sites in Detroit for NO_x_ and PM_2.5_. Note that both soft mean and soft variance capture the temporal variability depicted from the measurements, and that soft variance in Detroit is consistently low. Note also that R_ZeroOut/Total_ is constant in time.

Ultimately, both soft data (air quality observations from nonbackground sites adjusted using R_ZeroOut/Total_) and hard data (air quality observations from sites classified as background and located >60 km away from the local sources in Detroit) are then used by the STOK method to estimate background concentrations. We compared the distributions without outliers of hourly background sites, hourly soft data mean, and hourly STOK background for 2010 (Figure S1). For both NO_x_ and PM_2.5_, it is clear that the AQS soft data have a broader distribution (larger interquartile ranges, as well as higher differences between the 95th and 5th percentiles) compared to values from the background sites. However, when both datasets are combined through STOK, the distribution of the background concentrations estimated at the STOK estimation receptors (for e.g., locations of study participants) is comparable to the distribution of the AQS background sites for both pollutants. It is of note that PM_2.5_ depicts a median closer to actual observed sites classified as background than does NO_x._ This occurs because PM_2.5_ has several monitors classified as background in the vicinity of the STOK estimation receptors (Figure 2), whereas the NO_x_ monitors classified as background are farther away from the STOK estimation receptors (*i.e.*, the Detroit region). This further demonstrates how STOK estimates are influenced to a greater extent by hard data than soft data.

**Figure 9:**
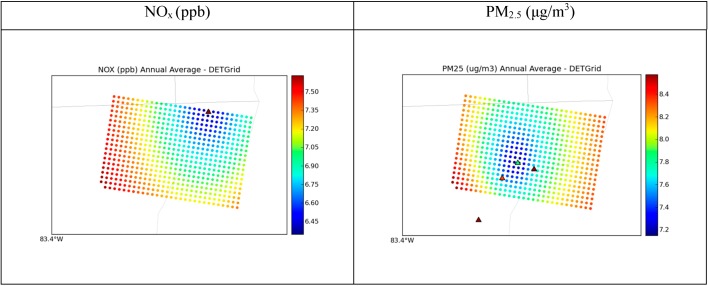
Spatial plots for the Detroit 30-km × 20-km grid showing annual averages of observed concentrations at AQS sites as triangles, and annual background concentration estimates from STOK as circles. Scales were adjusted to the range of background values to portray spatial variability of STOK estimation.

**Figure 10:**
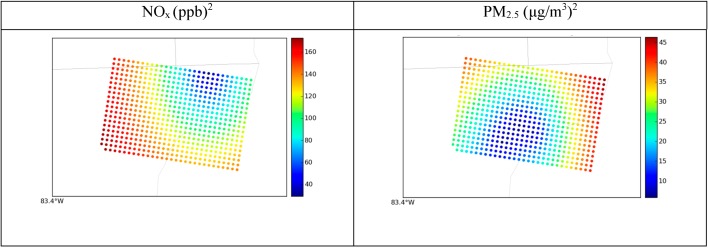
Spatial plots for the Detroit 30-km × 20-km grid showing annual averages of error variances from STOK as circles.

The spatial concentration gradient for the estimated background is shown in Figure 9. Because the AQS measurements shown are affected by local sources, they show significantly higher concentrations than the background concentrations. Note that the overall range of estimated background concentrations is very small, and the color scheme is chosen to capture the ranges of background concentrations and thus illustrate the spatial texture that STOK provides, and that may be important for accurate exposure estimates. NO_x_ has background estimates that range from around 6.5 ppb to 7.5 ppb and PM_2.5_ ranges from 7.2 μg/m^3^ to 8.4 μg/m^3^. However, these plots show how the concentration gradient from nonbackground monitors using soft data directly affects background concentrations for both pollutants. NO_x_ concentrations are affected by the northeast site, as can be seen from the spatial gradient propagating from the site. Some sites have more influence on background concentrations than others, as can be seen from the spatial gradient surrounding the AQS site in the middle of the domain, which influences PM_2.5_ concentrations more than the rest of the sites do. Figure 10 shows the error variance of STOK estimate, which measures the uncertainty of the estimates and helps identify areas with unreliable estimates. The error variance is high in the areas where no sampling data was available and low near the data points (*i.e.*, monitoring stations). The properties of kriging with measurement errors are such that the kriging estimation error variance is zero at the hard data points (*i.e.*, at background monitoring stations) and small but non-zero at the soft data points (*i.e.*, at non-background monitoring stations).

To evaluate the STOK method developed here, we performed two separate tasks. In the first, we evaluated estimated background concentrations at AQS monitoring locations that were designated background (see Supplemental Information Section 4 Table S3), and in the second, we evaluated the total hybrid concentrations against observed AQS concentrations in the Detroit region. For the latter, we aggregated background estimates (from our STOK methodology) with stationary and on-road concentrations (from AERMOD and R-LINE, respectively) to obtain the total concentration estimate (termed *new hybrid*) at each receptor. We also aggregated the total concentration from an earlier iteration of STOK with stationary and on-road concentrations (termed *old hybrid*); in the *old hybrid* approach, the adjustment of STOK estimates using CMAQ-based R_ZeroOut/Total_ was not used. These hybrid concentrations from the *old hybrid* and *new hybrid* approaches were paired in time and space and compared to available AQS sites (Figure 3) in the Detroit metro domain. 

**Figure 11:**
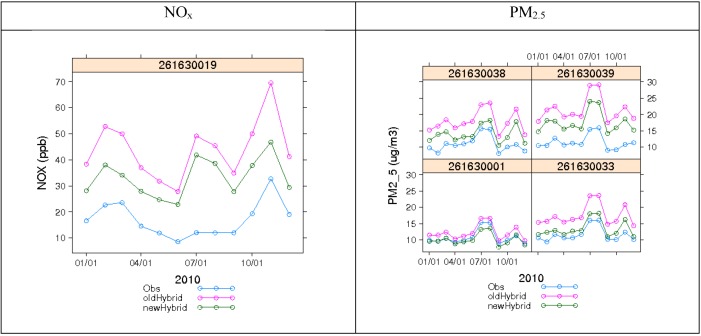
Time series plots showing monthly averaged observed concentrations paired spatially and temporally against hybrid estimates from the two STOK methods, one using all observed measurements as hard data (*old hybrid*) and the other using observations that were either soft or hard depending on the classification as background or not (*new hybrid*).

**Figure 12:**
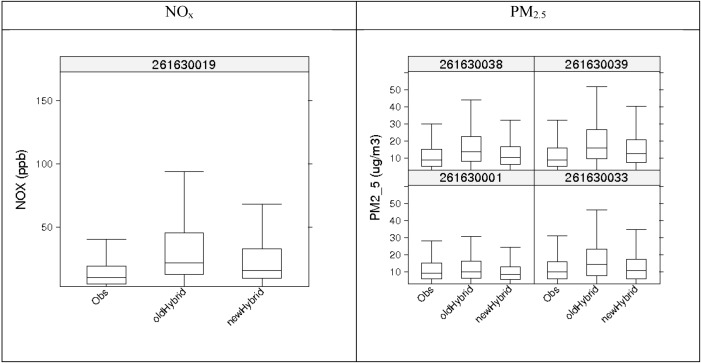
Comparison of distributions of hourly averaged observations paired spatially and temporally against hybrid estimates from the two STOK methods, one using all observed measurements as hard data (*old hybrid*) and the other using observations that were either soft or hard depending on the classification as background or not (*new hybrid*).

Figure 11 shows 2010 monthly average concentrations for observations, *old hybrid,* and *new hybrid* at each AQS site location. Both NO_x_ and PM_2.5_ depict an improvement in total concentration estimation due to lower and more accurate background estimates. This was expected, because at AQS sites the predicted background is equal to the actual observation. Most sites still slightly overpredict NO_x_ and PM_2.5_. NO_x_ site 261630019 (E 7 Mile Road) shows the worst performance in the summer, with estimates differing from observations by more than a factor of 2. A culprit for overprediction could be the overestimation from the stationary and on-road concentrations. The distributions of the hourly total concentrations for the same metrics (Figure 12) for NO_x_ and PM_2.5_ also show the same behavior—some overprediction with overall better performance with the improved STOK method that uses the CMAQ-based R_ZeroOut/Total_.

Figure S2 in Section 3 of the Supplementary Material shows the estimated background concentrations for NO_x_ and PM_2.5_ at the locations of the NEXUS study participants. This figure shows the detailed spatial texture that one is able to obtain from the STOK approach. 

## 5. Limitations and Future Work

Although the estimated concentration based on the STOK method showed good agreement with observed background concentration at AQS monitors (Figure 9, Figure 10, Figure 11 and Figure 12), the approach presented here has some limitations. First, the total concentration Z was assumed to be sum of the concentration from local source, Z_LS_, and the background concentration B. While this linearization of air pollution processes is a good approximation for dispersion processes, it may not capture some non-linear processes. Second, we assumed that a random variable *R*_Background/Total_ was normally distributed with a mean *μ_R_* and variance *σ*^2^*_R_* obtained from the output of two CMAQ simulations. The use of Gaussian distributions is needed because STOK is a linear geostatistical estimator that can only process Gaussian data. However, *R*_Background/Total_ can take values only between 0 and 1. Even though the soft data based on *R*_Background/Total_ improved the model performance, the distribution bounded between 0 and 1 could be considered in future work by using a non-linear geostatistical framework. 

Although the estimated concentration based on the STOK method showed good agreement with observed background concentration at AQS monitors (Figure 9, Figure 10, Figure 11 and Figure 12), the approach presented here has some limitations. First, the total concentration Z was assumed to be sum of the concentration from local source, Z_LS_, and the background concentration B. While this linearization of air pollution processes is a good approximation for dispersion processes, it may not capture some non-linear processes. Second, we assumed that a random variable was normally distributed with a mean and variance obtained from the output of two CMAQ simulations. The use of Gaussian distributions is needed because STOK is a linear geostatistical estimator that can only process Gaussian data. However, can take values only between 0 and 1. Even though the soft data based on improved the model performance, the distribution bounded between 0 and 1 could be considered in future work by using a non-linear geostatistical framework. 

The strength of this method relies on performing two CMAQ simulations with and without the zero-out sources. However, we recognize that this is a potentially resource-intensive task and hence a key limitation, and future efforts could focus on streamlining this process. Another limitation of our work is that urban background concentrations can seldom be measured directly, because it would require shutting down the urban local sources so that the background concentration with local sources zeroed out could be measured. Shutting down local sources in an urban area would in many cases be impractical, or even unethical. As a result the hard data on background concentrations are usually only available away from the local sources, which lack the specificity needed to conduct a traditional validation analysis within the urban area of interest (see Supplemental Information Section 4).

## 6. Summary and Conclusions 

Here we presented a novel method to estimate background concentrations of air pollutants in urban areas and its application to roadways in Detroit, Michigan, in support of the NEXUS urban-scale exposure and epidemiological study of the exposures to traffic-related pollutants of asthmatic children living near major roadways. Within a statistical framework, we estimated background concentrations at a very fine scale by leveraging data from routine but sparsely located monitors that are intended for capturing regional background concentrations along with other monitoring data that we subsequently adjusted using ratios computed from CMAQ—a detailed chemistry-transport model. 

We first explored the spatial and temporal characteristics of the available ambient monitoring data for a 3-yr monitoring period from 2009 to 2011 consistent with health data used in the epidemiological analysis. Results indicate the heterogeneity of the air pollutant concentration fields in space and time. Most of the variability in the concentrations of the regional pollutant studied here (PM_2.5_) can be attributed to longer-term synoptic-scale patterns and short-term fluctuations (such as morning and afternoon peaks in traffic). Thus, regional pollutants such as PM_2.5_ are spatially homogeneous but temporally heterogeneous. On the other hand, traffic-related pollutants (e.g., NO_x_) are both spatially and temporally heterogeneous. This is because of the significant contribution of local sources, especially for monitors located near major roadways. As indicated by the analysis of observational data, air quality modeling tools need to account for all major multiscale atmospheric processes, including both local impacts (associated with local-scale variations of emissions and meteorology) and regional impacts (background levels associated with synoptic patterns). 

Although evaluation of the combined STOK product with observations reveals an overprediction of PM_2.5_ and NO_x_, the estimated background concentrations closely mirror the observed time series in most cases. In addition, the STOK method improves the characterization of background concentrations for both PM_2.5_ and NO_x_ as compared to a technique that does not account for double-counting. 

In conclusion, we have demonstrated that our novel technique combining air quality observations from sites that are designated as background along with ratios computed from detailed air quality models can eliminate previous issues related to double-counting, provide detailed spatial coverage, and provide a viable approach to characterizing background air quality in urban areas in support of environmental health studies.

## Supplementary Files

Supplementary File 1
