# Supplementary material for: A Method for Estimating Urban Background Concentrations in Support of Hybrid Air Pollution Modeling for Environmental Health Studies
Source: Int J Environ Res Public Health. 2014 Oct 15;11(10):10518–36. doi: 10.3390/ijerph111010518 (PMC4210993; doi:10.3390/ijerph111010518)
Supplement: Supplementary File 1 [file ijerph-11-10518-s001.pdf]

Supplementary Information

A Method for Estimating Urban Background Concentrations in Support of Hybrid Air Pollution Modeling for Environmental Health Studies

---

1. The 64 Unique Monitoring Locations Used in This Study for Estimating Background Concentrations

Table S1. AQS sites used in the STOK estimation, along with monitor objective (background sites shown in red).

Name	State	AIRS-ID	NO_x	$PM_{2.5}$	Both	Monitor Objective
Pittsboro Elementary School	IN	180630002	✓			Highest Concentration
Old Ammunition Bunker	IN	180890022	✓			Highest Concentration
Naval Avionics Center	IN	180970073	✓			Population Exposure
Washington Park	IN	180970078		✓		Population Exposure
Ernie Pyle School	IN	180970081		✓		Population Exposure
South Bend - Shields Dr.	IN	181410015	✓			Population Exposure
Fire Station #17	IN	181630012	✓			Highest Concentration
Evansville- Buena Vista	IN	181630021	✓			Highest Concentration
McLean High School	IN	181670018		✓		Unknown
Bay City	MI	260170014		✓		Unknown
Whaley Park	MI	260490021		✓		General Background
Lansing	MI	260650012			✓	Unknown
Kalamazoo Fairgrounds	MI	260770008		✓		Population Exposure
GR-Monroe	MI	260810020		✓		Population Exposure
Tecumseh	MI	260910007		✓		General Background
Houghton Lake	MI	261130001			✓	Unknown/General Background
Port Huron	MI	261470005		✓		General Background
Seney	MI	261530001		✓		General Background
Ypsilanti	MI	261610008		✓		Population Exposure
Allen Park	MI	261630001		✓		Population Exposure
East 7 Mile	MI	261630019	✓			Unknown
Dearborn Public Schools	MI	261630033		✓		Unknown
Newberry School	MI	261630038		✓		Unknown
FIA/Lafayette	MI	261630039		✓		Unknown
Adams	OH	390010001		✓		Unknown
Lima Bath	OH	390030009		✓		Unknown
Athens	OH	390090004	✓			Regional Transport
Hook Field Airport	OH	390171004		✓		Population Exposure
Springfield Firehouse	OH	390230005		✓		Unknown
GT CRAIG	OH	390350060	✓			Population Exposure
New Albany	OH	390490029		✓		Unknown

Table S1. *Cont.*

Name	State	AIRS-ID	NO_x	$PM_{2.5}$	Both	Monitor Objective
Columbus State Fairgrounds	OH	390490034		✓		Unknown
Yellow Springs	OH	390570005		✓		Unknown
Sycamore	OH	390610006		✓		Unknown
Steuben	OH	390810017		✓		Source Oriented
Odot	OH	390870012		✓		Population Exposure
Erie	OH	390950024		✓		Unknown
Youngstown	OH	390990014		✓		General Background
Chippewa	OH	391030004	✓			Upwind Background
Dayton Public Library	OH	391130032		✓		Population Exposure
National Trail School	OH	391351001		✓		Regional Transport
Health Dept.	OH	391510020		✓		Population Exposure
Laird	OH	391550005		✓		Unknown
Lebanon	OH	391650007		✓		Unknown
Narsto Site Arendtsville	PA	420010001			✓	Extreme Downwind
Lawrenceville	PA	420030008			✓	Population Exposure
Carnegie Science Center	PA	420030010	✓			Population Exposure
South Allegheny School	PA	420030064		✓		Population Exposure
Harrison	PA	420031005	✓			Population Exposure
Kittanning	PA	420050001		✓		Unknown
Beaver Falls	PA	420070014	✓			Population Exposure
Reading Airport	PA	420110011	✓			Population Exposure
Bristol	PA	420170012	✓			Population Exposure
Johnstown	PA	420210011	✓			Population Exposure
State College (PSU)	PA	420270100	✓			Population Exposure
Harrisburg	PA	420430401	✓			Population Exposure
Chester	PA	420450002	✓			Population Exposure
Marne	PA	420490003	✓			Population Exposure
Scranton	PA	420692006	✓			Population Exposure
Lancaster	PA	420710007	✓			Population Exposure
Freemansburg	PA	420950025			✓	Population Exposure
Perry County	PA	420990301	✓			General Background
Charleroi	PA	421250005	✓			Population Exposure
York	PA	421330008	✓			Population Exposure

Note: * Houghton Lake is designated background for $PM_{2.5}$ but not for NO_x.

2. The Covariance Model, and the Two Components for Each of NO_x and $PM_{2.5}$

In this work, we developed the geostatistical framework for the space/time estimation of ambient concentration of air pollutants. Because of its ability to produce not only the estimate at unmonitored locations but also the uncertainty associated with the estimate, the geostatistical method known as kriging has been widely used in air quality studies. Here, we employed the method of space/time ordinary kriging (STOK) with measurement error to estimate the ambient concentration at any unmonitored location in the study area.

The space/time dependency of each air quality parameter was characterized by the means of its covariance function (covariogram). We assumed that each air quality parameter had a constant global offset given by the simple arithmetic average of all the observations in the study domain, and that the residuals obtained by subtracting this constant global offset from the observations were isotropic and homogeneous-stationary. The latter assumption implies that the space/time covariance function of the residuals depends solely on the spatial and temporal distance between two space/time points.

The space/time sample covariance function of the residual concentrations was modeled by the method-of-moments estimator $\hat{C}$ at various spatial lag r and temporal lag τ. The sample covariance function was then used to fit a positive-definite covariance model. In this work, we used the space/time separable two-component exponential covariance model defined as

$$C(r,\tau) = C_1 \exp\left(-\frac{3r}{a_{r1}}\right)\exp\left(-\frac{3\tau}{a_{\tau1}}\right) + C_2 \exp\left(-\frac{3r}{a_{r2}}\right)\exp\left(-\frac{3\tau}{a_{\tau2}}\right)$$

where C_1 and C_2 are the sill parameters quantifying the variability of observations, a_{r1} and a_{r2} are spatial ranges, and $a_{\tau1}$ and $a_{\tau2}$ are temporal ranges for the 1st and 2nd components. The range parameter characterizes the extent of the influence of spatial and temporal autocorrelation and is given by the separation distance at which the covariance decreases to 5% of the sill. First we obtained the spatial component of the sample covariance function at temporal lag $\tau = 0$ and the temporal component at spatial lag $r = 0$. These sample covariance functions were then used to fit the model. All covariance model parameters were estimated by an automated weighted least squares procedure. Table S2 shows the estimated parameters of the space/time separable two-component exponential covariance model defined by the above equation.

Table S2. Covariance model parameter for $PM_{2.5}$ and NO_x obtained by the weighted least squares method. C_1 and C_2 are the sill parameter, a_{r1} and a_{r2} are spatial range, and $a_{\tau1}$ and $a_{\tau2}$ are temporal range for the 1st and 2nd component, respectively.

Pollutant	1st Component			2nd Component		
	C_1	a_{r1}	$a_{\tau1}$	C_2	a_{r2}	$a_{\tau2}$
**$PM_{2.5}$**	69.2728	1.2044	0.83387	34.5523	62.5145	24.3498
**NO_x**	214.1213	1.3139	0.73731	48.5727	693.0062	765.778

The STOK with measurement error method was employed to obtain the estimate and associated estimation variance at each estimation point. The STOK estimate is given by the linear combination of nearby samples. The kriging weights are obtained by minimizing the estimation mean square error subject to the unbiasedness constraint. In this study, we used 50 nearby samples that were observed within five temporal units (days or hours) from the estimation time. Nearby samples were selected based on the space/time distance defined as

(space/time distance) = (spatial distance) + ((space/time metric) × (temporal distance))

where (space/time metric) is the ratio of the spatial covariance range and temporal covariance range. The STOK with measurement error method was implemented in the Matlab R2012a (MathWorks Inc., Natick, MA, USA) and *BMElib* libraries for modern spatiotemporal geostatistics.

3. Background Concentrations for NEXUS

Figure S1. Distribution of observations with background monitor objectives, soft data mean from nonbackground monitors, and STOK estimation for NO_x (left) and $PM_{2.5}$ (right) for Detroit 30-km × 20-km grid of receptors with outliers removed (showing median as red line, 25th and 75th percentiles as ends of the boxes, and 5th and 95th percentiles as whiskers).

NO_x (ppb)	$PM_{2.5}$ (µg/m^3)

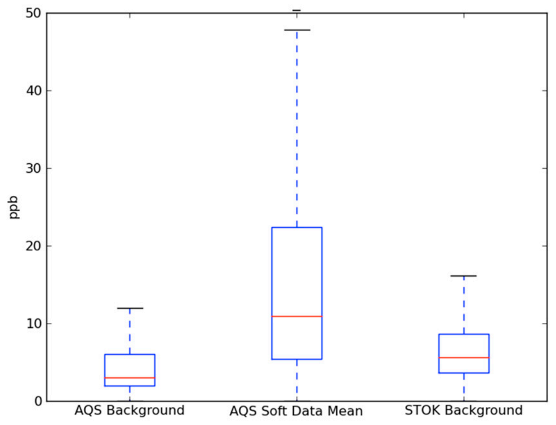

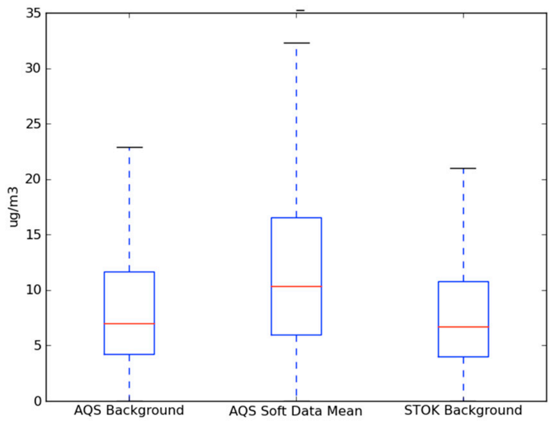

Figure S2. Estimated NO_x and $PM_{2.5}$ background concentrations using STOK for the NEXUS study locations.

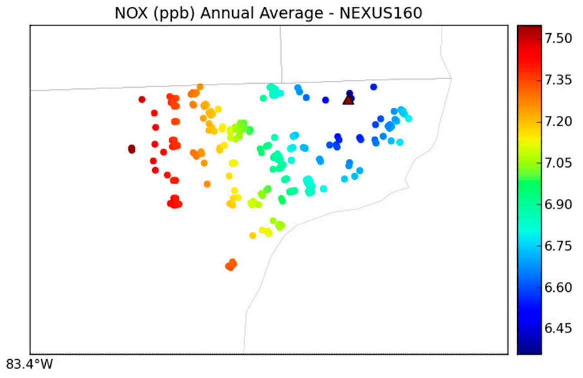

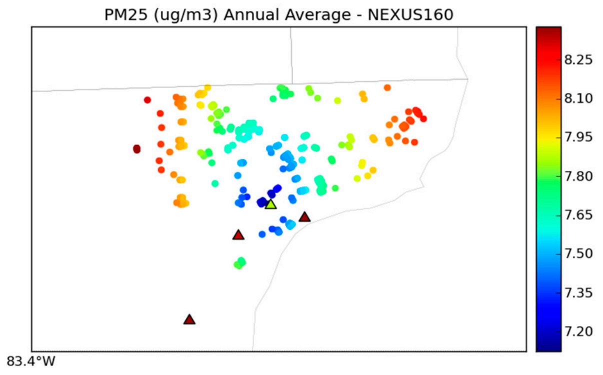

4. STOK Method Validation

A challenging aspect of our work is that urban background concentrations can seldom be measured directly, because it would require shutting down the urban local sources so that the background concentration with local sources zeroed out could be measured. As a result the hard data on background concentrations are usually only available away from the local sources, which lack the specificity needed to conduct a traditional validation analysis within the urban area of interest.

To examine this issue, we attempted to perform a validation analysis using a validation dataset consisting of measurements obtained at sites located outside of Detroit and with a background monitoring objective (two sites for NO_x and six sites for $PM_{2.5}$, as identified in Table S1, and shown in Figure 2). We implemented two methods, where we exclude the validation background sites and only utilize non-background sites as the training dataset for estimation.

1. The "Old Method" consists of using non-background sites as hard data, thus not employing the CMAQ-based $R_{ZeroOut/Total}$, and
2. The "New Method" which consists of using soft data at the non-background site locations. The latter method employs both observations at non-background sites and the CMAQ-based $R_{ZeroOut/Total}$.

Both methods estimate hourly background concentrations for 2010 for $PM_{2.5}$ and NO_x. Table S3 shows statistical metrics that compare both methods to observations.

Table S3. Statistical metrics comparing observed measurements at background sites against STOK estimates using only non-background sites as hard data points (Old Method) and using only soft data from non-background sites (New Method) for NO_x and $PM_{2.5}$ for 2010 (Units are in ppb for NO_x and μg/m^3 for $PM_{2.5}$ for means and % for all error statistics).

Metric	NO_X		$PM_{2.5}$	
	**Old Method**	**New Method**	**Old Method**	**New Method**
Observed Mean	4.41	4.41	8.96	8.96
Model Mean	28.38	12.14	10.25	9.71
Mean Error	23.97	7.73	1.29	0.74
Mean Absolute Error	24.02	8.52	3.14	3.17
Root Mean Squared Error	37.23	11.93	4.81	4.92
FAC2 *	6%	22%	83%	83%

Note: * FAC2 represents the percent of concentrations estimates within a factor of two of observations.

NO_x results show ~60% reduction of the model mean from the old to the new method. This translates to a general improvement of NO_x model performance as seen by our metrics shown in Table S3. For instance we see that the Old Method has a large positive Mean Error +23.97%, and this Mean Error reduces to +7.73% for the New Method, which demonstrates that the large over prediction of the Old Method (which does not correct for double counting) is reduced in the New Method (which corrects for double counting). Likewise we see a reduction of the Mean Absolute Error and a reduction in the Root Mean Square Error between the Old and New Methods, demonstrating that on average the New Method is successful at reducing the absolute and the squared error compared to the Old Method. Another measure of goodness is the FAC2, which is the coverage of observed values by a factor of 2 around the estimated values. The FAC2 increases from 6% to 22%, demonstrating a substantial improvement from the Old to the New Method.

$PM_{2.5}$, on the other hand, shows a model mean reduction of only ~5%, and even though model performance is far better for $PM_{2.5}$ (compared to NO_x), the same improvement is not seen when comparing the two methods. It shows a negligible model performance difference between methods across most metrics. Hence overall, our validation analysis shows that the New Method is better than the Old Method for NO_x, but in the case of $PM_{2.5}$ the difference is too close to be conclusive.

This behavior confirms that, in the case of $PM_{2.5}$, our validation dataset lacks the specificity needed to conduct a traditional validation analysis within the urban area of interest. The difference in the specificity of the validation data between NO_x and $PM_{2.5}$ can be attributed to the difference in the spatial ranges of their covariance models. As shown in Table S2, $PM_{2.5}$ has spatial range components (a_{r1}, a_{r2}) of 1.2 km and 62 km. This means that the $PM_{2.5}$ validation background stations need to be within 62 km of Detroit if they are to see an influence from the training data points located in Detroit. These training data points are treated as hard by the Old Method (thereby not correcting for double counting), whereas in the New Method they are treated as soft data with a CMAQ-based correction factor of $R_{ZeroOut/Total}$. Since the $PM_{2.5}$ validation background stations are outside the range of influence of the local sources, they lack the specificity needed to distinguish between the New and Old Methods. NO_x, on the other hand, has spatial range components of 1.3 km and 693 km. Since there are NO_x validation background stations that fall within 693 km of Detroit, these validation stations provide data with the specificity needed to distinguish between the new and old methods.

In conclusion, our validation shows how method 2 (New Method) improves NO_x estimation compared to method 1 (Old Method) because the NO_x validation background stations were specific enough to Detroit (e.g., within the NO_x covariance range of 693 km). On the other hand a limitation of our study is that it lacks $PM_{2.5}$ background stations that are specific enough to Detroit to distinguish method 1 from method 2 (*i.e.*, the $PM_{2.5}$ background stations were all outside the covariance range of 62 km around Detroit). As a result the validation is not able to assess what would be the improvement in estimation accuracy for $PM_{2.5}$ background concentrations if we were able to measure $PM_{2.5}$ background concentrations close to Detroit. To truly measure background at Detroit we would have to shut down local sources in the city, which would be a difficult, and in many cases, unethical task. Thus, we choose the path of comparing hybrid estimation (by combining local-scale dispersion model results with estimated background) against observations in the urban monitors in Detroit.

© 2014 by the authors; licensee MDPI, Basel, Switzerland. This article is an open access article distributed under the terms and conditions of the Creative Commons Attribution license (http://creativecommons.org/licenses/by/4.0/).
